# MR imaging biomarkers for evaluating therapeutic effects shortly after near infrared photoimmunotherapy

**DOI:** 10.18632/oncotarget.7357

**Published:** 2016-02-12

**Authors:** Yuko Nakamura, Marcelino Bernardo, Tadanobu Nagaya, Kazuhide Sato, Toshiko Harada, Peter L. Choyke, Hisataka Kobayashi

**Affiliations:** ^1^ Molecular Imaging Program, Center for Cancer Research, National Cancer Institute, Bethesda, MD, USA; ^2^ Research Technology Program, SAIC-Frederick Inc., National Cancer Institute, Bethesda, MD, USA

**Keywords:** near infrared photoimmunotherapy, MRI, T2 relaxation time, diffusion weighted image, gadofosveset

## Abstract

Near infrared photoimmunotherapy (NIR-PIT) is a new cancer treatment that combines the specificity of antibodies for targeting tumors with the toxicity induced by photon absorbers after irradiation with NIR light. The purpose of this study was to determine if MR imaging can detect changes in the MR properties of tumor within several hours of NIR-PIT. A431 cells were injected subcutaneously in the right and left dorsi of 12 mice. Six days later, the mice were injected with a photon absorber, IR700, conjugated to panitumumab, an antibody targeting epidermal growth factor receptor. One day later, only right sided tumor was exposed to NIR light (treated tumor). MRI was performed 1 day before and 1-2 hours after NIR-PIT using gadofosveset for six mice and gadopentetate dimeglumine for another six mice. T2 relaxation times, the apparent diffusion coefficient (ADC) for the following combinations of b-values: 0-1000, 200-1000 and 500-1000 s/mm^2^ and enhancement indices were compared before and after NIR-PIT using a two-sided paired *t*-test. For treated tumors, T2 relaxation time increased after NIR-PIT (p < 0.01) and all three ADC values decreased after NIR-PIT (*p* < 0.01). Moreover, the enhancement area under the curve (AUC) using gadofosveset increased after NIR-PIT (*p* = 0.02). In conclusion, prolongation of T2, reductions in ADC and increased enhancement using gadofosveset are seen within 2 hours of NIR-PIT treatment of tumors. Thus, MRI can be a useful imaging biomarker for detecting early therapeutic changes after NIR-PIT.

## INTRODUCTION

Near infrared photoimmunotherapy (NIR-PIT) is a newly developed cancer treatment that employs a targeted monoclonal antibody conjugated to a photon absorber, IRDye700DX (IR700, silica-phthalocyanine dye) [1]. The first-in-human phase I trial of NIR-PIT in patients with inoperable head and neck cancer targeting epidermal growth factor receptor was approved by the US FDA, and is underway as of June 2015 (https://clinicaltrials.gov/ct2/show/NCT02422979).

In this trial, a patient is injected with an antibody- photon absorber conjugate (APC) that binds to target molecules on the cell membrane of the tumor. About 24 hours later the tumor is exposed to NIR light at a wavelength of 690 nm which is absorbed by the dye. This induces nearly immediate necrotic cell death rather than apoptotic cell death which is induced by most other cancer therapies. Within minutes, cells treated with NIR-PIT rapidly increase in volume leading to rupture of the cell membrane, and extrusion of cell contents into the extracellular space [2-5]. In contrast, cells dying by apoptosis tend to shrink without membrane disruption over a period of days [6-8]. Moreover, because the APC tends to preferentially bind to the layers of cells in the immediate perivascular space, subsequent NIR-PIT leads to perivascular tumor cell death thereby promoting increases in vascular permeability permitting even nano-sized particles to enter the treated tumor beds [9]. The dramatic increase in permeability for nanoparticles, followed by their retention in NIR-PIT treated tumors has been termed ‘super-enhanced permeability and retention (SUPR)’. SUPR effects induced by NIR-PIT have been reported to allow 5-15 fold increases in accumulation of the liposomal anti-cancer agent (DaunoXome®) resulting in more effective therapy than either NIR-PIT or liposomal anti-cancer agent alone. These changes occur within 20 min of NIR light exposure, yet gross tumor size and shape do not change for several days after NIR-PIT.

It is important to establish clinically applicable imaging biomarkers for rapidly evaluating the effects of NIR-PIT as this could be used to direct additional NIR light if the initial therapeutic effect is not satisfactory. Magnetic resonance imaging (MRI) is a widely used imaging modality that demonstrates both anatomic and functional information in a short time. T2 relaxation times and apparent diffusion coefficients (ADC) can be determined from relatively routine imaging sequences not requiring contrast media injection. These quantitative parameters may provide useful insights into the physiologic changes that occur following NIR-PIT. Moreover, to assess vascular permeability there are several types of commercially available MR contrast agents, both low and high molecular weight. Gadofosveset (MS-325; Ablabar®; Lantheus Medical Imaging, North Bilerica, Ma, USA) is a small molecular weight Gd-chelate that reversibly binds albumin making it a macromolecular MR blood pool agent [10-12]. Gadofosveset-enhanced MRI has also been reported to be useful in the assay of tumor angiogenesis and tumor capillary permeability [13, 14]. Therefore, gadofosveset-enhanced MRI could be useful for evaluation of SUPR effects following NIR-PIT.

The purpose of this study was to determine whether MR imaging parameters such as T2, ADC and dynamic contrast enhancement may be useful in evaluating the immediate therapeutic effects of NIR-PIT.

## RESULTS

### T2 relaxation time and ADC value

**Figure 1:**
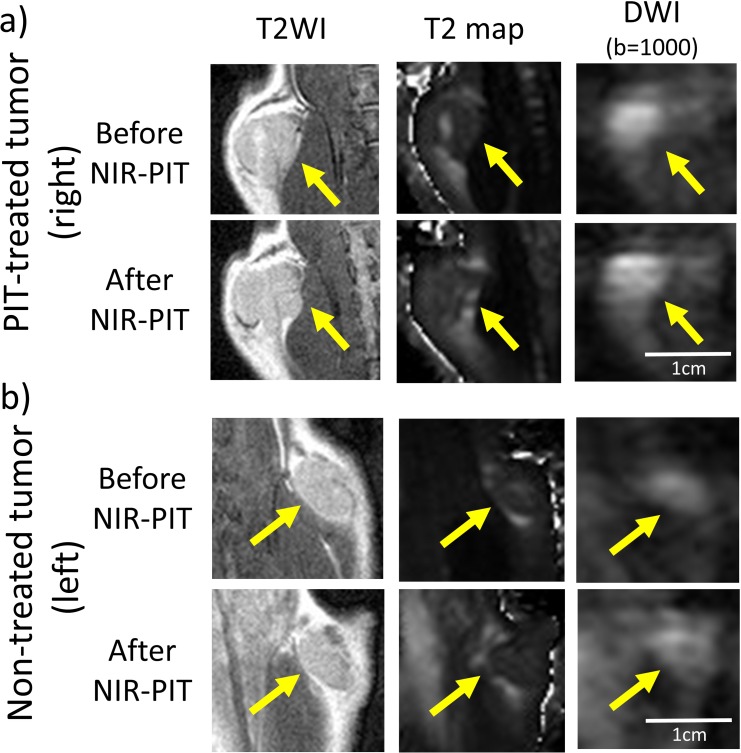
T2WI, T2 map, and DWI with b-value of 1000 sec/mm^2^ of a NIR-PIT-treated **a.** or a non-treated control tumor **b.** before and after NIR-PIT. Arrow indicates the tumor.

Post treatment T2 relaxation times and the three ADC values are shown in Table 1. T2 relaxation time of treated tumors (right sided tumors) increased after NIR-PIT (*p* < 0.01). However, T2 relaxation time of non-treated tumors (left sided tumors) decreased after NIR-PIT (*p* = 0.01). After NIR-PIT the three ADC values, calculated from different pairs of b values, decreased in treated tumors (*p* < 0.01). Interestingly, non-treated tumors also exhibited a decline in ADC values for one but not all b value pairings. For instance for the b = 0 and 1000 s/mm^2^ pair, a decline in ADC was seen (*p* = 0.04) but was not seen with the combinations of b = 200 and 1000 (*p* = 0.13) or b = 500 and 1000 s/mm^2^ (*p* = 0.07) (Figure 1).

**Table 1:** T2 relaxation time and apparent diffusion coefficient (ADC) values

	Before NIR-PIT	After NIR-PIT	*P* value
PIT-treated tumors			
T2 relaxation time (ms)	181.94 ± 34.04	219.37 ± 30.05	<0.01
ADC value (x 10^−3^) b=0 and 1000 sec/mm^2^	1.21 ± 0.26	1.02 ± 0.23	<0.01
ADC value (x 10^−3^) b=200 and 1000 sec/mm^2^	1.17 ± 0.26	0.98 ± 0.21	<0.01
ADC value (x 10^−3^) b=500 and 1000 sec/mm^2^	1.15 ± 0.27	0.86 ± 0.30	<0.01
Non-treated tumors			
T2 relaxation time (ms)	214.51 ± 24.68	195.94 ± 15.80	0.01
ADC value (x 10^−3^) b=0 and 1000 sec/mm^2^	1.22 ± 0.23	1.14 ± 0.23	0.04
ADC value (x 10^−3^) b=200 and 1000 sec/mm^2^	1.16 ± 0.26	1.08 ± 0.20	0.13
ADC value (× 10^−3^) b=500 and 1000 sec/mm^2^	1.14 ± 0.29	0.97 ± 0.23	0.07

*Data are mean ± standard deviation.

T2 relaxation time and ADC values before and after NIR-PIT

T2 relaxation time of treated tumors (right sided tumors) increased after NIR-PIT (p < 0.01). However, T2 relaxation time of non-treated tumors (left sided tumors) decreased after NIR-PIT (p = 0.01). After NIR-PIT the three ADC values, calculated from different pairs of b values, decreased in treated tumors (p < 0.01). Non-treated tumors also exhibited a decline in ADC values for one but not all b value pairings.

### Changes observed with dynamic gadofosveset- enhanced MRI

**Figure 2:**
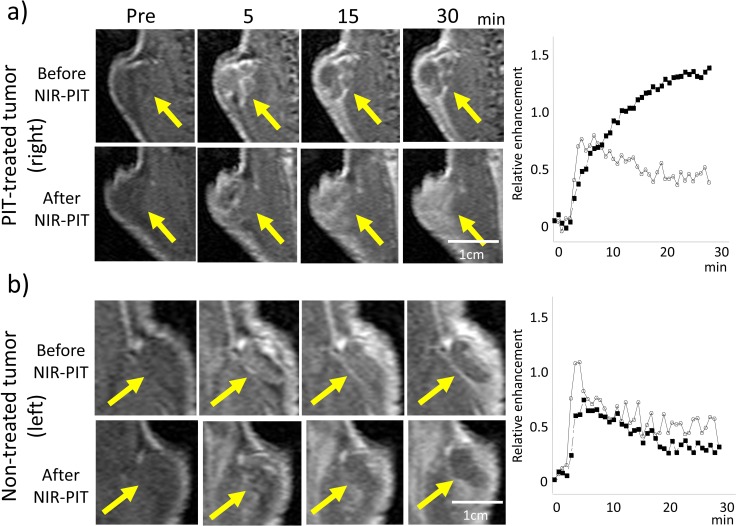
Dynamic MR images after injection of gadofosveset in a NIR-PIT-treated **a.** or non-treated control tumor **b.** before and after NIR-PIT (pre-injection, and 5, 15, and 30 min after gadofosveset injection). Arrow indicates the tumor. Before NIR-PIT the treated tumor enhanced immediately after gadofosveset injection and contrast enhancement decreased gradually. After NIR-PIT contrast enhancement of the treated tumor increased gradually until 30 min after gadofosveset injection. On the other hand, contrast enhancement of the non-treated tumor after NIR-PIT seemed to decrease slightly compared to the tumor before NIR-PIT. Time enhancement curve of the NIR-PIT-treated tumor (a: right panel) and the non-treated tumor (b: right panel) using gadofosveset (before (open circles) and after NIR-PIT (filled squares)).

The effects of NIR-PIT on Dynamic contrast enhanced (DCE) MRI using gadofosveset are shown in Table 2. For treated tumors area under the curve (AUC) increased after NIR-PIT (*p* = 0.02) (Figure 2a). However, the other parameters showed no change. For non-treated tumors maximum relative enhancement (MRE) and AUC decreased after NIR-PIT (*p* = 0.02, and 0.03, respectively) (Figure 2b).

**Table 2:** Indices of enhancement after dynamic study with gadofosveset

	Before NIR-PIT	After NIR-PIT	*P* value
PIT-treated tumors			
MRE	0.90 ± 0.24	1.10 ± 0.20	0.11
TTP	446.43 ± 368.18	862.60 ± 741.56	0.26
WIR	0.40 ± 0.22	0.52 ± 0.51	0.57
WOR	0.03 ± 0.01	0.02 ± 0.02	0.69
AUC	20.19 ± 7.64	28.02 ± 6.35	0.02
Non-treated tumors			
MRE	1.17 ± 0.13	0.90 ± 0.20	0.02
TTP	272.40 ± 49.73	454.00 ± 319.74	0.20
WIR	0.54 ± 0.20	0.39 ± 0.30	0.07
WOR	0.04 ± 0.01	0.03 ± 0.01	0.09
AUC	24.33 ± 6.81	20.50 ± 6.45	0.03

*Data are mean ± standard deviation.

Indices of enhancement after dynamic enhanced study with gadofosveset before and after NIR-PIT

Relative enhancement (RE) was calculated using the equation: RE = [SI(D)/SI(Dref)-1], where SI(D) stands signal intensity (SI) of dynamic current and SI(Dref) stands for the initial SI. The RE curve of the ROI was determined from a series of images. Maximum RE (MRE) is the maximum RE observed during the entire DCE MRI. Time to peak (TTP) is the time between the beginning of the injection (T_0_) and the MRE. Wash-in rate (WIR) (RE/second) is the maximum slope between T_0_ and MRE (designated as T_1_). Wash-out rate (WOR) (RE/second) is the maximum slope between T_1_ and the end of the measurement. Area under the curve (AUC) is the area measured under the time-RE curve.

For treated tumors AUC increased after NIR-PIT (p = 0.02). For non-treated tumors MRE and AUC decreased after NIR-PIT (p = 0.02, and 0.03, respectively).

### Changes observed with dynamic gadopentetate dimeglumine-enhanced MRI

**Figure 3:**
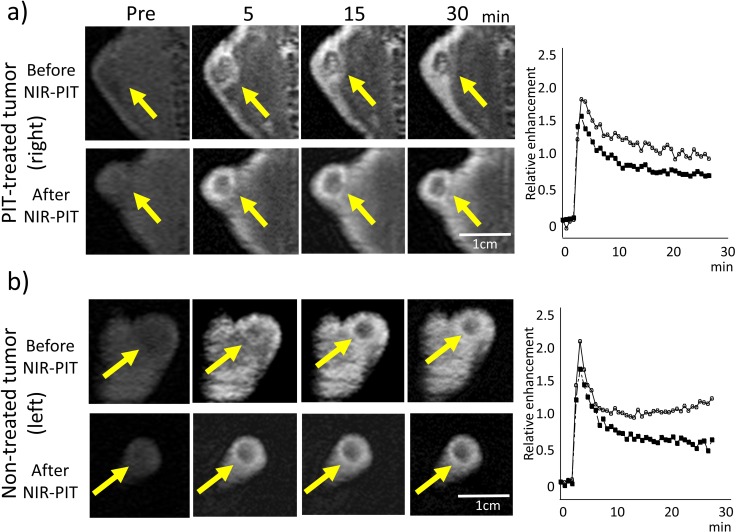
Dynamic MR images after injection of gadopentetate dimeglumine in the NIR-PIT-treated **a.** or non-treated control tumor **b.** before and NIR-PIT (pre-injection and 5, 15, and 30 min after gadopentetate dimeglumine injection). Arrow indicates the tumor. Before NIR-PIT the treated tumor enhanced immediately after gadopentetate dimeglumine injection and contrast enhancement decreased gradually. Contrast enhancement did not change before and after NIR-PIT. Contrast enhancement of the non-treated tumor also seemed not to change before and after NIR-PIT. Time enhancement curve of the NIR-PIT-treated tumor (a: right panel) and non-treated control tumor (b: right panel) using gadopentetate dimeglumine (before (open circles) and after NIR-PIT (filled squares)).

The effects of NIR-PIT on dynamic MRI using gadopentetate dimeglumine are shown in Table 3. No significant differences were observed in either treated or non-treated tumors using this contrast agent (Figure 3).

**Table 3:** Indices of enhancement of dynamic study with gadopentetate dimeglumine

	Before NIR-PIT	After NIR-PIT	*P* value
PIT-treated tumors			
MRE	1.59 ± 0.40	1.51 ± 0.39	0.65
TTP	522.1 ± 574.8	506.97 ± 580.28	0.81
WIR	0.52 ± 0.34	0.49 ± 0.29	0.81
WOR	0.06 ± 0.02	0.06 ± 0.02	0.80
AUC	34.18 ± 10.30	31.96 ± 12.33	0.68
Non-treated tumors			
MRE	1.50 ± 0.40	1.52 ± 0.51	0.91
TTP	332.93 ± 136.70	218.26 ± 33.98	0.10
WIR	0.61 ± 0.21	0.76 ± 0.38	0.41
WOR	0.06 ± 0.03	0.07 ± 0.03	0.41
AUC	28.98 ± 7.43	24.35 ± 7.37	0.31

*Data are mean ± standard deviation.

Indices of enhancement after dynamic enhanced study with gadopentetate dimeglumine before and after NIR-PIT

Relative enhancement (RE) was calculated using the equation: RE = [SI(D)/SI(Dref)-1], where SI(D) stands signal intensity (SI) of dynamic current and SI(Dref) stands for the initial SI. The RE curve of the ROI was determined from a series of images. Maximum RE (MRE) is the maximum RE observed during the entire DCE MRI. Time to peak (TTP) is the time between the beginning of the injection (T_0_) and the MRE. Wash-in rate (WIR) (RE/second) is the maximum slope between T_0_ and MRE (designated as T_1_). Wash-out rate (WOR) (RE/second) is the maximum slope between T_1_ and the end of the measurement. Area under the curve (AUC) is the area measured under the time-RE curve.

No significant differences were observed in either treated or non-treated tumors.

### Pathological analysis

**Figure 4:**
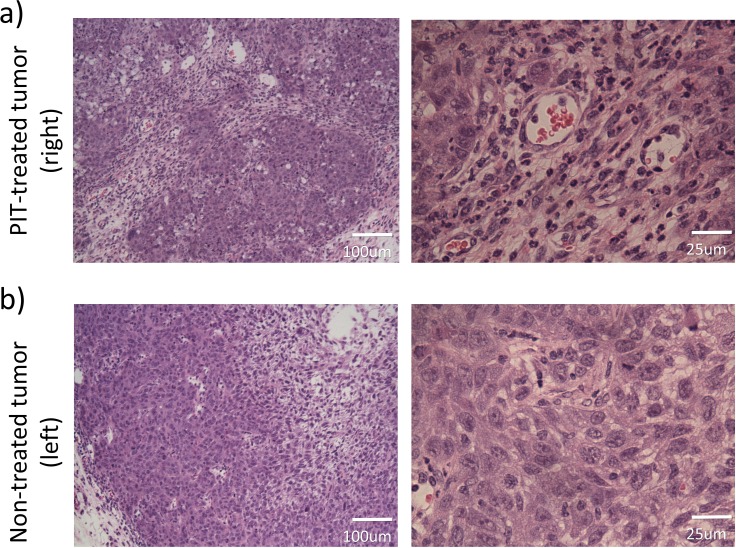
Histological analyses of the NIR-PIT-treated **a.** or non-treated control tumor **b.** (hematoxylin and eosin staining). NIR-PIT induced large areas of perivascular necrotic cell death. The perivascular tumor cells were severely damaged **a.**. In non-treated control tumor necrosis was limited to the center of the tumor. Perivascular tumor cells did not show any damage **b.**.

In treated tumors, intensive necrotic cell death in perivascular areas was confirmed, coinciding with the pathological result of NIR-PIT treated tumor in which SUPR was observed [9] (Figure 4a). In non-treated tumors, central necrosis was also confirmed. However, the location of necrosis was limited to the center of the tumor probably because of insufficient vascular supply due to rapid growth and high central interstitial pressures. Perivascular tumor cells did not show any damage (Figure 4b).

## DISCUSSION

This study demonstrates that NIR-PIT induces rapid changes in the MR properties of tissues that can be readily measured using standard MR pulse sequences available on practically all MRI units. For instance, T2 relaxation time was increased after NIR-PIT probably because of rapid cell volume expansion leading to increased water content in tissue and edema in tumor bed due to increases of vascular permeability [2, 3]. Similarly, it is likely that cell expansion and the increased viscosity of the extracellular space due to cell rupture after NIR-PIT leads to lowering water diffusion. Moreover, vascular permeability, as measured by increased retention of the macromolecular gadofosveset (when bound to albumin) as compared to the lack of increased retention with the low molecular weight gadopentetate dimeglumine immediately after NIR-PIT, appears to increase after NIR-PIT. Thus, NIR-PIT causes profound and early changes in common MR properties of treated tissues.

Changes in MR properties were also observed to a much lesser degree in the non-treated tumors. While it is tempting to speculate regarding a systemic effect of treatment on one side affecting tumors on the other side a more prosaic may underlie these observations. The tumor model used in this study, A431 is a very fast growing tumor and significant changes can occur even within one day. For instance, the T2 relaxation time of the non-treated tumors consistently decreased slightly after contralateral NIR-PIT. The A431 cell line proliferates very rapidly compared with cancers in patients, so that cellularity of the tumor might increase within a day leading to a decrease in free water and hence, a decrease in T2 relaxation time. Similarly, whereas the treated tumors demonstrated an increase in permeability to gadofosveset, non-treated tumors showed a decrease in permeability after contralateral NIR-PIT. It is common for this tumor model to rapidly develop necrosis as the tumor attains diameters of 7-8 mm, because tumor growth outstrips vascular supply. Therefore, these minor decreases in permeability could be explained by rapid growth of A431 cells *in vivo*.

A somewhat more puzzling result is the changes in ADC after NIR-PIT. Tissue perfusion-related and true molecular diffusion can be evaluated separately with the intravoxel incoherent motion (IVIM) model [15]. The effect of perfusion on signal decay on DWI with b-values greater than 200 has been reported to be quite small [16, 17]. Thus, for evaluation of true molecular diffusion we calculated ADC values using b = 200 and 1000, and b = 500 and 1000 sec/mm^2^. There was substantially more noise using the b = 200 and 500 sec/mm^2^ images compared to b = 0 sec/mm^2^ images and this could influence the apparent changes on ADC. From our results, the ADC was also lower for non-treated tumors but only with one of the three b value pairings (b = 0 and 1000 sec/mm^2^). This effect may be due to tumor growth in the interval causing increased water restriction. The decreases in ADC in treated tumors are more difficult to explain and somewhat surprising given that most treatments result in increases in ADC. One might expect the breakdown of cell membranes and rupture of cells to result in increased water diffusion with higher ADC values due to improved opportunities for water diffusion. However, at least early on, ADC values were lower in treated tumors regardless of how it was calculated, indicating that true molecular diffusion was more restricted in treated tumors compared to that of non-treated tumors. It is possible that cell expansion and increases in viscosity due to the sudden extrusion of intracellular contents after cell rupture leads to decreased water diffusion, at least early after NIR-PIT [18]. Further investigation of this phenomenon is planned.

Gadofosveset is a low molecular weight chelate that quickly and reversibly binds serum albumin allowing the protein-bound gadofosveset to remain largely intravascular. T1-weighted MR imaging enhanced with macromolecular contrast medium (MCCM) has been shown to be useful in identifying hyper-permeable micro-vessels in tumors. Circulating blood levels of MCCM remain almost constant for several hours, while the tumor interstitium gradually accumulates macromolecules leaking through the hyperpermeable endothelium [19, 20]. Gadofosveset reversibly binds to albumin and therefore is “mostly” a macromolecular contrast agent but does exist as a small molecule agent for a portion of its lifetime and therefore is not entirely macromolecular. Thus, in its albumin-bound form, gadofosveset-enhanced MRI can potentially yield information on both perfusion and hyper-permeability of vessels. From our results, NIR-PIT induced increased maximum relative enhancement whereas non-treated tumors demonstrated a decrease in maximum relative enhancement. While the latter likely reflects necrosis, the former is likely due to the SUPR effect that has been seen with other macromolecules after NIR-PIT. This is best explained by understanding that the cells most heavily exposed to the APC are located in perivascular space and their death, after NIR-PIT, leads to a dramatic reduction in vascular resistance and therefore increases in flow and leakage/retention.

On the other hand, the low molecular weight gadopentetate dimeglumine enhanced dynamic MRI revealed no differences before and after NIR-PIT, either in the treated or non-treated tumor. As a small molecule there is a certain amount of leakage of gadopentate dimeglumine expected in tumors. The fact that NIR-PIT did not induce significant increases in enhancement suggests that perfusion is relatively constant before and after NIR-PIT, whereas leakage of low molecular weight agents, which is already high is not further increased by NIR-PIT. Thus, the SUPR effect, as reflected by gadofosveset but not gadopentate dimeglumine, is mostly confined to macromolecular agents.

In addition to MRI, other imaging methods of assessing NIR-PIT are possible. For instance CT perfusion study or ^18^F-FDG PET study could be used to evaluate the SUPR effect with direct cell killing effect. We have previously shown that ^18^F-FDG PET can depict the acute cytotoxic effects of NIR-PIT due to shutting down the glucose metabolism [21]. With X-ray CT perfusion, only small molecular contrast agents similar to gadopentetate dimeglumine are available. In this study, we evaluated macromolecular MR contrast agents or protein binding agents required for evaluating SUPR effects. Additionally, X-ray CT perfusion study requires repeated scanning resulting in high radiation exposure to patients. Fluorescence imaging is also an important modality for evaluation of micro residual disease after NIR-PIT [22-32]. However, the utility of fluorescence imaging is limited clinically because it cannot image deep inside the body. On the other hand, MR imaging is widely used and can evaluate any part of the body regardless of the depth. Therefore, our result using MR can be transferred to the clinic easily.

Our MRI data was not correlated with other treatment effects, such as tumor growth or overall survival of the animals. This is the first study evaluating acute effects of NIR-PIT with MRI. Therefore, in this work, we focused on determining the appropriate MR imaging methods for evaluating early NIR-PIT effects. Although we have already fully studied NIR-PIT therapeutic effects with this tumor model in previous studies [1, 2], correlation with MRI findings is worthwhile future project.

Previous reports suggested that the light emitting diode (LED) light source for NIR-PIT should be standardized because light dose may be affected by distance and temperature [33]. However, these issues are mitigated by using a laser. In this study we performed NIR-PIT with 689 ± 4 nm NIR laser light through a parallel collimator (500 mW/cm^2^, duration 40 sec). Therefore, light dose was minimally affected by distance compared with LED light sources. We also confirmed the power output as 500 mW/cm^2^ by using an optical power meter using the same settings as our experiment including distance between laser and target. Moreover, we always use an electric fan to dissipate heat at the surface.

In conclusion, NIR-PIT induces increases in T2 relaxation time and increases in enhancement with gadofosveset but not gadopentate dimeglumine. Decreases in ADC are also seen likely due to dramatic change in cell volume and tissue viscosity as a result of rapid cellular necrosis. These MR imaging findings are potentially important imaging biomarkers for evaluating the immediate treatment effects of tumors treated with NIR-PIT.

## MATERIALS AND METHODS

### Cell lines and culture

A431 cells expressing human epidermal growth factor receptor 1 (HER1) were grown in RPMI1640 supplemented with 10 % FBS and 1 % penicillin-streptomycin in tissue culture flasks in a humidified incubator at 37°C in an atmosphere of 95 % air and 5 % carbon dioxide.

### Reagents

Water soluble, silica-phthalocyanine derivative, IRDye700DX NHS ester was obtained from LI-COR Bioscience (Lincoln, NE, USA). Panitumumab, a fully humanized IgG2 monoclonal antibodies (mAb) directed against HER1, was purchased from Amgen (Thousand Oaks, CA, USA). All other chemicals were of reagent grade.

### Synthesis of IR700-conjugated panitumumab

Conjugation of dyes with mAb was performed according to previous reports [1, 3, 9]. In brief, panitumumab (1 mg, 6.8 nmol) was incubated with IR700 NHS ester (60.2 μg, 30.8 nmol) in 0.1 mol/L Na_2_HPO_4_ (pH 8.6) at room temperature for 1 hour. The mixture was purified with a Sephadex G25 column (PD-10; GE Healthcare). The protein concentration was determined with Coomasie Plus protein assay kit (Thermo Fisher Scientific Inc) by measuring the absorption at 595 nm with spectroscopy (8453 Value System; Agilent Technologies). The concentration of IR700 was measured by absorption at 689 nm with spectroscopy to confirm the number of fluorophore molecules conjugated to each mAb. The synthesis was controlled so that an average of three IR700 molecules were bound to a single antibody. We abbreviate IR700 conjugated to panitumumab as Pan-IR700.

### Animal model

All procedures were performed in compliance with the Guide for the Care and Use of Laboratory Animals [34] and approved by the local Animal Care and Use Committee. Twelve female homozygote athymic nude mice with six- to 8-week old, average weight 27 g, were purchased from Charles River (National Cancer Institute Frederick). A431 cells (2 × 10^6^ in phosphate-buffered saline) were injected subcutaneously in the right and left dorsi of the mice under isoflurane anesthesia.

### Near infrared photoimmunotherapy

Pan-IR700 (100 μg per 200 μl diluted with phosphate-buffered saline) was injected intravenously at 6 days after injection of the tumor cells. Fluorescence images were obtained with a Pearl Imager (LI-COR Biosciences) at 24h after injection of Pan-IR700 and immediately after NIR-PIT. The tumors on the right dorsum were NIR light exposed with NIR laser light at 685 to 693 nm wavelength (BWF5-690-8-600-0.37; B&W TEK INC., Newark, DE, USA) (500 mW/cm^2^ x 40 s, 20 J/cm^2^) while the left sided tumors and the remainder of the body were shielded from light with aluminum foil. The power density of light in mW/cm^2^ was measured with an optical power meter (PM100, Thorlabs, Newton, NJ, USA). This dose of 20 J/cm^2^ by NIR laser light was selected because it has been shown to reliably induce SUPR effects. 20J/cm^2^ induced by laser light is equivalent to 50 J/cm^2^ NIR light emitted by LED in this tumor model [9].

### MRI

#### Imaging techniques

Under isoflurane anesthesia animals were scanned 1 day before NIR-PIT and 1-2 hours after NIR-PIT. This timing was selected because the animals needed to be transferred to the MRI suite and because SUPR has been previously visualized 1-6 hours after NIR-PIT [9]. MRI was performed on a 3-T scanner using an in-house 10 inch saddle shaped mouse receiver coil array (Intera Acieva 3T; Philips Medical Systems, Best, Netherlands). Scout images were obtained to accurately locate the tumor. All mice underwent T2-weighted imaging (T2-WI), T2-mapping, and diffusion weighted imaging (DWI). The parameters for T2-WI were TR/TE 4000 ms/30 ms, echo train length 6, flip angle (FA) 90, field-of-view (FOV) 80 × 40 mm, matrix 400 × 198, pixel size 0.2 × 0.2 mm, slice thickness and gap 0.6/0 mm, acquisition time 4 min and 32 s, and number of slices 40. For T2 mapping a multi-echo sequence was employed as follows: TR 3000 ms, TE 22, 33, 44, 55, 66, 77, 88, 99, 110, 121, 132, 143, 154, 165, 176, 187 ms, echo train length 16, FA 90, FOV 80 × 40 mm, matrix 132 × 69, pixel size 0.61 × 0.59 mm, slice thickness and gap 1/0 mm, acquisition time 4 min and 30 s, and number of slices 24. For DWI, the parameters were TR 5748 ms, TE 77 ms, echo train length 9, FA 90, FOV 80 × 40 mm, matrix 80 × 30, pixel size 1.0 × 1.3 mm, slice thickness and gap 1/0 mm, number of excitations 1, b-values: 0, 100, 200, 500 and 1000 s/mm^2^, acquisition time 4 min and 1 s, and number of slices 24.

DCE MRI was performed with T1-weighted spoiled gradient-echo sequence (SPGR) every 45.4 seconds over a 30-minute period. After acquiring 3 baseline scans 0.3 mmol/kg of gadofosveset was infused in six mice or 0.2 mmol/kg of gadopentetate dimeglumine (Magnevist, Berex, Wayne, NJ) was infused in 6 other mice over 1 minute. The SPGR parameters were TR 15 ms, TE 2.2 ms, FA 24, FOV 64 × 32 mm, matrix 320 × 80, pixel size 0.2 × 0.4 mm, slice thickness and gap 0.5/0 mm, number of excitations 1, acquisition time 45.4 s, and number of slices 48.

All images were obtained in the coronal plane. Total acquisition time was about 45 min.

#### Image analysis

All images were analyzed using Image J software (http://rsb.info.nih.gov/ij/). Both right (treated) and left (non-treated) tumors were chosen as the regions of interest (ROIs). First, T2 relaxation times were measured on T2 maps. Next, ADC was calculated using the formula ADC = ln [−Sx/Sy]/(bx − by), and utilizing a pair of b values; Sx is the signal intensity (SI) at a given b-value of x s/mm2 and Sy is the b-value of y s/mm2, based on the mono-exponential model. We calculated three ADC values based on 3 pairs of b values (b = 0 and 1000, 200 and 1000, 500 and 1000 s/mm^2^) for each ROI. For the dynamic contrast enhanced study, we converted SI to relative enhancement (RE) using the equation: RE = [SI(D)/SI(Dref)-1], where SI(D) stands SI of dynamic current and SI(Dref) stands for the initial SI. The RE curve of the ROI was determined from a series of images. Next, we calculated five parameters: maximum RE (MRE), time to peak (TTP), wash-in rate (WIR), wash-out rate (WOR), and area under the curve (AUC). MRE is the maximum RE observed during the entire DCE MRI. TTP is the time between the beginning of the injection (T0) and the MRE. WIR (RE/second) is the maximum slope between T0 and MRE (designated as T1). WOR (RE/second) is the maximum slope between T1 and the end of the measurement. AUC is the area measured under the time-RE curve.

### Pathological analysis

To evaluate histological changes shortly after NIR-PIT, light microscopy was performed using an Olympus BX61 microscope (Olympus America, Inc., Melville, NY). A431 tumors were harvested in 10 % formalin after the 2^nd^ MR examination. Serial 10 μm slice sections were fixed on glass slides with hematoxylin and eosin staining.

### Statistical analysis

Statistical analysis was performed with JMP 10 software (SAS Institute, Cary, NC). To compare all MR parameters before and after NIR-PIT the two-sided paired *t*-test was employed. Differences of *p* < 0.05 were considered statistically significant.
